# Liposomes for Enhanced Bioavailability of Water-Insoluble Drugs: In Vivo Evidence and Recent Approaches

**DOI:** 10.3390/pharmaceutics12030264

**Published:** 2020-03-13

**Authors:** Mi-Kyung Lee

**Affiliations:** Department of Pharmaceutical Sciences, Woosuk University, Jeonbuk 55338, Korea; leemk@woosuk.ac.kr; Tel.: +82-63-290-1423

**Keywords:** liposomes, oral, absorption, water-insoluble drugs, bioavailability

## Abstract

It has been known that a considerable number of drugs in clinical use or under development are water-insoluble drugs with poor bioavailability (BA). The liposomal delivery system has drawn attention as one of the noteworthy approaches to increase dissolution and subsequently absorption in the gastrointestinal (GI) tract because of its biocompatibility and ability to encapsulate hydrophobic molecules in the lipid domain. However, there have been several drawbacks, such as structural instability in the GI tract and poor permeability across intestinal epithelia because of its relatively large size. In addition, there have been no liposomal formulations approved for oral use to date, despite the success of parenteral liposomes. Nevertheless, liposomal oral delivery has resurged with the rapid increase of published studies in the last decade. However, it is discouraging that most of this research has been in vitro studies only and there have not been many water-insoluble drugs with in vivo data. The present review focused on the in vivo evidence for the improved BA of water-insoluble drugs using liposomes to resolve doubts raised concerning liposomal oral delivery and attempted to provide insight by highlighting the approaches used for in vivo achievements.

## 1. Introduction

Drug discovery and development are remarkably complex and challenging because numerous attributes should be simultaneously optimized to achieve clinically desirable efficacy and safety. In particular, there has been growing emphasis on drug-like properties such as solubility and permeability being considered in the early phase of drug discovery and development [1]. According to the Biopharmaceutical Classification System (BCS), BCS class II and IV drugs show low oral BA. For BCS class II drugs, low BA can be mainly due to poor dissolution. In contrast, the low BA of BCS class IV drugs is caused by both poor dissolution and low permeability [1]. Diverse approaches have been attempted to deliver water-insoluble drugs: salt formation, co-solvency and surfactant solubilization, amorphous forms, solid dispersion, co-crystals, polymeric micelles, inclusion complex, size reduction, solid lipid nanoparticles, polymeric nanoparticles and liposomes [2,3,4]. Particulate delivery systems, including liposomes, have drawn attention because they can solubilize water-insoluble drugs into the nano-sized structure and modulate in vivo behavior of the drug to reduce toxicity [5]. Among the nanoparticles, liposome has been one of the most extensively tried systems because it is biocompatible enough to be approved for parenteral administration [6].

**Figure 1:**
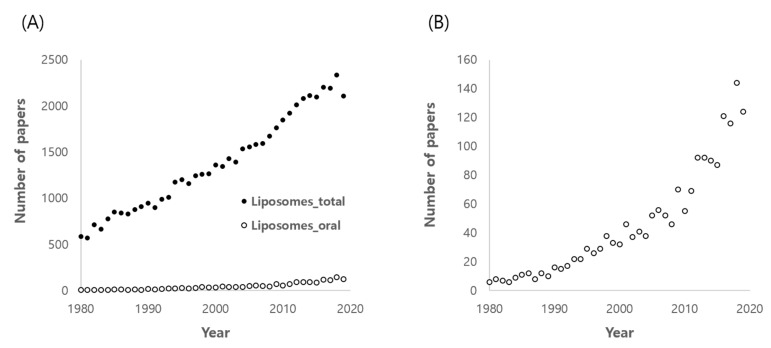
Number of papers published from the years 1980 to 2000. The numbers were obtained from a Pubmed search using keywords of liposomes (Liposomes_total) or liposomes and oral (Liposomes_oral). (**A**) represents both results, ‘Liposome_total’ and ‘Liposomes_oral’, for comparison. (**B**) represents ‘Liposomes_oral’ only to show the increasing trend.

Liposomes, vesicles enclosed by phospholipid bilayers, can solubilize water-insoluble drugs into the lipid domain of the liposomal membrane [5,7]. In addition to their solubilizing capacity and biocompatibility, the structural and compositional similarity of liposomes with bio-membranes has also encouraged their application for non-invasive oral delivery of poorly-permeable drugs [8]. However, there have been several drawbacks to be overcome, such as instability in the GI tract and poor permeability across the intestinal epithelia because of liposomes’ relatively large size [8]. Moreover, it seemed that liposomal oral delivery was faltering during 1980s due to some disappointing results for insulin [9]. However, liposomal oral delivery resurged with a rapid increase in the number of papers published, as shown in Pubmed search results, mainly due to various advanced modification technologies, even though liposomal oral delivery-related papers account for only 5–6% of the total number of liposomes-related studies (Figure 1). In addition, there was an encouraging meta-analysis report in which phospholipid-based solid formulations were analyzed as being effective for BA enhancement [10]. Although liposomal delivery does not seem to achieve satisfactory improvement of oral BA for peptides and protein drugs yet, it looks more promising for oral delivery of hydrophobic drugs because liposomes can solubilize poorly water-soluble drugs, protect the drug from degradation in the GI tract and enhance permeability through the epithelial cell membrane, consequently increasing oral BA. However, most published papers performed in vitro studies only, thus lacking in vivo pharmacokinetic results. The present review focuses on the in vivo evidence for the improved BA of water-insoluble drugs and highlights the approaches used for in vivo achievements.

## 2. Overview of Liposomes as Drug Delivery System

### 2.1. Basic Composition and Structure 

**Figure 2:**
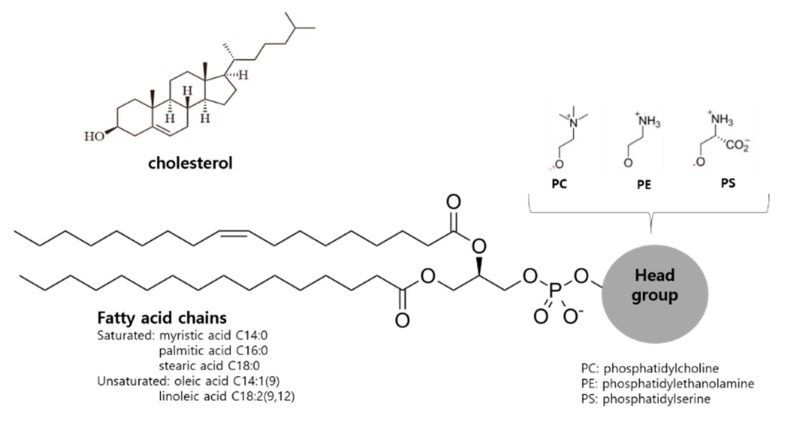
Structure of phospholipids and cholesterol.

**Figure 3:**
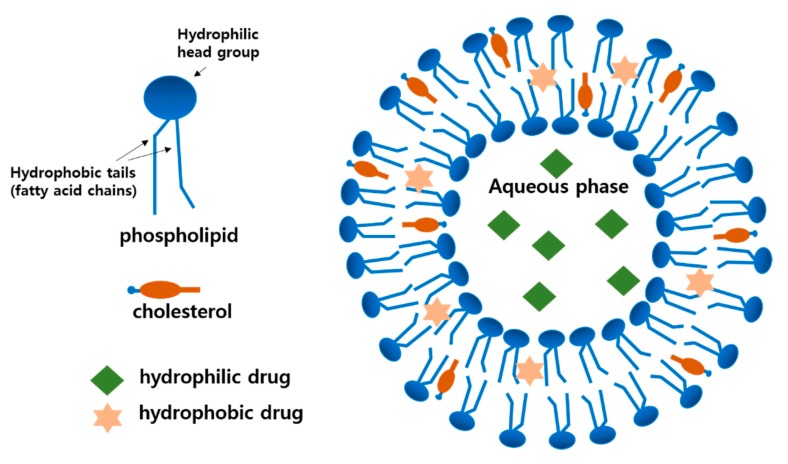
Structure of conventional liposome encapsulating hydrophilic and hydrophobic drugs.

Liposomal membranes are analogous to the cell membranes composed of phospholipids bilayers. Phospholipids can spontaneously form vesicles upon hydration with aqueous media due to the amphiphilic molecular structure possessing a hydrophilic phosphatidyl head group and hydrophobic fatty acid tails, as shown in Figure 2. Cholesterol (CH) can also be easily incorporated into the liposomal membrane in the same manner as the plasma membrane and stabilize the membrane modulating drug release, as presented in Figure 3 [6]. As shown in the structure, hydrophilic drugs can be encapsulated into the inner aqueous phase, while hydrophobic drugs reside in the lipid tail domain of the bilayer. Similarly to biomembranes, liposomal membranes are fluidic; hence, the encapsulated drugs can be released by or leaked out of the liposomes. The release rate has been known to be dependent on the composition of liposomal membranes, such as the presence of CH and the type of fatty acid acyl chains of the phospholipids [11]. 

### 2.2. Preparation Methods 

**Figure 4:**
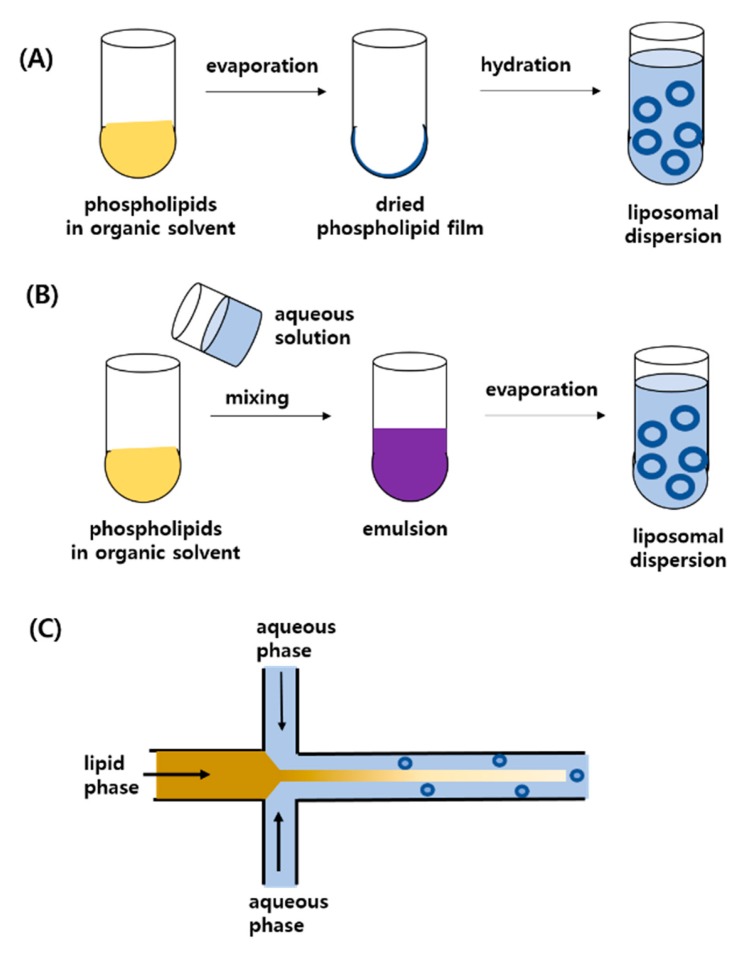
Representative techniques for the preparation of liposomes: film hydration method (**A**), reverse phase evaporation method (**B**) and microfluidic method (**C**)**.**

Liposomes can be spontaneously formed upon hydration of phospholipids, as mentioned above. The conventional method of preparing liposomes is the film hydration method. As depicted in Figure 4, the first step is to dissolve the phospholipids into organic solvents, such as chloroform, methanol, ether and others, followed by drying by evaporation of the organic solvent to obtain thin lipid film. The lipid film can be hydrated with aqueous media, such as water, buffers or saline solution to obtain liposomal dispersion. In general, multilamellar vesicles (MLVs) enclosed by multiple bilayers are formed through the film hydration method. This dispersion can then be subjected to a further homogenization step such as sonication or high-pressure homogenization to reduce particle size and obtain a more homogenous size distribution. The other classical method is the reverse-phase evaporation technique in which emulsion is formed by mixing the phospholipids solution in organic solvents with aqueous phase followed by the removal of organic solvent using rotary evaporator to obtain liposomal dispersion. However, the two above-mentioned classical methods are not suitable for the mass production of liposomes. In recent years, microfluidic technology has been developed to prepare in a single step for clinical and industrial application. As simply depicted in Figure 4, the microfluidic methods can provide well controlled mixing of the phospholipids solution with aqueous media, providing more precise control of particle size, charge and surface modification [7,12]. Although there are several other techniques, the present review does not cover full details for the numerous other preparation methods because it is beyond the scope of the present review and most of them are basically analogous to the above-mentioned techniques. 

To encapsulate drugs in the preparation process, hydrophobic drugs are solubilized with lipid in organic solvent, while hydrophilic drugs are added into aqueous media for hydration. As depicted in Figure 4, hydrophobic drugs may reside lipid domains of the liposomal membrane, while hydrophilic drugs are encapsulated inside the aqueous phase. It should be also noted that not all the drugs are encapsulated into liposomes, which requires to separate the free drug from the encapsulated ones. In general, free drugs can be removed from the liposomes by dialysis, ultracentrifuge or size-exclusion chromatography [13,14]. In nanomedicine for water-insoluble drugs, it has been a challenge to increase the drug loading efficiency, the mass fraction of drug in the entire drug-loaded nanoparticles. It has been reported that various nanoparticles showed only 10% or much lower loading efficiency [15]. As shown in Table 1, the liposomal formulations for water-insoluble drugs exhibited 80–90% of encapsulation efficiency, the percentage of drugs encapsulated into liposomes compared to the initially loaded amount of drug. In general, as long as the optimal ratio of drug to lipids is maintained, the encapsulation efficiency of hydrophobic drugs into liposomes is high because water-insoluble drugs cannot be dissolved in hydration media and hence, almost all the drugs may be retained in the lipid domains. However, due to the limited space of the lipid domain in the liposomal membrane, the loading efficiency seldom exceeds 20–30%. Recently, Yang et al. showed that their fabrication platform of core-shell nanoparticles could promote the loading efficiency up to 65% [15]. For liposomes, this requires to develop or adopt more active loading technology to increase the loading efficiency of water-insoluble drugs. 

### 2.3. Evaluation of Liposomal Characteristics 

It has been known that small changes in liposomal formulations may significantly affect clinical outcomes such as pharmacokinetic and pharmacodynamics performances [46]. Kapoor et al. presented an effect of critical quality attributes (CQAs) on the in vivo performance of liposomes based on the FDA’s experience by reviewing liposomal products submitted for approval [14]. They insisted that characterization should be performed for CQAs for liposomal products such as particle size, particle size distribution, lipid impurities, in vitro drug release, lamellarity, free and encapsulated drug ratios, etc. As shown in Table 2, several major liposome-specific characteristics have been included in the studies of liposomes. Particle size and particle size distribution are key players for the efficacy of liposomal drug product modulating pharmacokinetics. A common technique for measuring particle size and size distribution is the dynamic light scattering (DLS) method. Additionally, morphology may be evaluated by electron microscopy, providing additional information on the particle size and lamellarity. Recently, a wide range of microscopy techniques has been applied to visualize liposomes, which includes polarization, fluorescence microscopy and various electron microscopy methods such as transmission, cryo, freeze fracture and environmental scanning electron microscopy [47]. In vitro release from the liposomes should be performed in an appropriate simulated physiological medium. In vitro release is critical to understand the in vivo behavior of the product because the encapsulated liposomal drug may show significantly different in vivo behavior from the released free drug [13,46]. FDA guidance encourages the establishment of in vitro and in vivo correlation in order to justify the use of an in vitro release test for the prediction of in vivo performance. In the same context, encapsulation efficiency is measured to optimize the formulation with maximum encapsulation. Liposomes are thermodynamically unstable, hence, prone to fusion or aggregation during storage. In most cases, the stability of liposomes is evaluated by the change in particle size. The FDA proposed that chemical stability of liposomal lipids should be evaluated as well. In addition, most liposome researchers evaluate surface charge by measuring zeta potential to understand liposome–liposome and liposome–membrane interactions. Charged liposomes can be prepared by incorporating anionic or cationic molecules into the liposomal membrane components or surface coating with charged polymers. Charged liposomes can be stabilized through electrostatic repulsion protecting fusion or aggregation between liposomes.

### 2.4. Advantages and Disadvantages of Liposomes for Oral Delivery

The advantages and disadvantages of liposomes as an oral delivery system are summarized in Table 3. The greatest advantage of liposomes as a drug delivery system may be their excellent biocompatibility, which has already been proven by the approved liposomal formulations for intravenous administration, such as Doxil^®^, a doxorubicin-containing stealth liposome. As mentioned above, liposomes can encapsulate various drugs, including hydrophilic and hydrophobic drugs, as well as peptides, proteins, and nucleic acids, without loss of activity [48]. There is a large variety of available phospholipids and other lipids providing enormous flexibility of formulations. It has been well known that various characteristics, such as encapsulation efficiency, drug release rate and stability can be controlled by varying compositions of phospholipids with diverse fatty acid chains. As proven in the commercial product, Doxil^®^, the liposomal surface is modifiable with polyethylene glycol chains which can be conjugated with targeting moieties such as folic acid and antibody [49]. The intrinsic similarity of the liposomal membrane to the cellular membrane has evoked the expectation that liposomes may enhance the permeation of drugs through the skin barrier and oral mucosal membrane. At the cellular level, liposomal drugs were taken up by cancer cells through endocytosis pathway, as opposed to the unencapsulated free drug, which can permeate the cellular membrane via simple diffusion [50]. It has been general knowledge that nanomedicine can modify the pharmacokinetics and biodistribution of drugs. For oral delivery, the release of the drug from the liposomes can be delayed or controlled by liposomal membrane formulation, leading to different absorption rates. Liposomes can lead the drug into the lymphatic pathway, bypassing the hepatic first-pass effect [51]. In addition, drug-induced GI irritation may be reduced by avoiding direct contact with the intestinal environment encapsulating into liposomes. 

Despite all the potential advantages, there have been several apparent limitations of liposomes for oral delivery. First, liposomes are unstable in the dispersed liquid state due to the inherent thermodynamic instability accompanied by particle size change and drug leakage during storage. In addition, the liposomal structure is not resistant to the acidic environment of the stomach. In the same manner as various nanoparticles, it is difficult for the intact liposomes to permeate freely across intestinal barriers due to their relatively large size [52]. One of the biggest limitations of liposomes as a drug delivery system has been the difficulty in continuous mass production and quality control because the liposomal system is very complex [8,14]. Lipid degradants such as lysolipids can be formed during manufacturing or storage. Lysolipids have been known to be associated with toxicity such as hemolysis and apoptosis and thus, the content should be monitored [14]. 

## 3. Current Approaches Used for In Vivo Studies

It was expected that liposomes had been applied and showed enhanced BA for a great number of water-insoluble drugs. However, only a limited number of drugs were found in in vivo studies using liposomes, as shown in Table 1. The present review tried to determine which strategies have been successful for the improving oral BA of water-insoluble drugs by reviewing the in vivo results listed in Table 1. In addition, the review includes not only liposomal dispersion in liquid form but also solid dosage forms, such as proliposomes, freeze-dried liposomes, solid dispersion and co-precipitate with phospholipid, which can form a vesicular structure upon hydration. 

### 3.1. Stabilization 

Conventional liposomes have been known to be unstable in the presence of gastric acid, bile salts and pancreatic lipase to release incorporated drugs [53,54,55,56]. The remnants from the disrupted liposomes can form mixed micelles with bile salts in the GI tract and the encapsulated water-insoluble drugs will be transferred and solubilized into the micelles [56]. The newly formed micelles can then transport the incorporated drugs to the epithelial cells for absorption. The premature release of water-insoluble drugs would results the precipitation or aggregation in the GI tract and subsequently, reduce the bioaccessibility, which is defined as the fraction of drug that is solubilized in the digesta and therefore, available for absorption [57]. In addition to the instability in the GI tract, poor stability during preparation and storage has been challenge in oral drug delivery using liposomes as well. Various strategies have been attempted to stabilize liposomes. 

#### 3.1.1. Modulation of Lipid Compositions 

In liposome research fields, phospholipids with a higher phase transition temperature (T_c_) than 37 °C have been regarded as more stable and sustained release carriers compared to those with lower T_c_ [58,59,60]. As shown in Table 1, several liposomes included high T_c_ phospholipids such as DMPC (24 °C, DPPC (41 °C), DSPC (55 °C) and HSPC (52 °C). However, low Tc phospholipids such as EPC (−5 to −15 °C) and SPC (−20 to −30 °C) have been widely used in combination with cholesterol and bile salts because they are easy to handle in the preparation process [7]. As seen in Table 1, the type of lipid hardly affects the oral BA of water-insoluble drugs. Nevertheless, most studies listed in Table 1 compared in vitro characteristics such as encapsulation efficiency and particle size among various compositions to select the optimal one in the preparation stage to proceed to the next in vivo absorption studies. 

#### 3.1.2. Formulation in Solid Forms

Proliposomes are dry and free-flowing powder which can form liposomes upon hydration with aqueous media and have been widely tried for oral delivery since being introduced by Payne et al. in 1986 [61]. Proliposomes provide various advantages over the conventional liposomes dispersed in aqueous media. In general, improved stability can be achieved due to their solid-state during storage. In addition, proliposomal powder can be incorporated into solid dosage forms such as tablet and capsule. Moreover, the preparation process is relatively straightforward for industrial scale manufacturing [62]. Proliposomes can be prepared by adsorbing phospholipids dispersed in organic solvent into carriers such as microcrystalline cellulose, mannitol, lactose or sorbitol and thereafter, drying under reduced pressure to remove excess solvent. The freeze-drying technique has been also used to obtain the solid form of liposomes. Liposomes can be freeze-dried in the presence of suitable cryoprotectants such as trehalose, mannitol or sucrose. From these solid-type liposomal formulations, liposomes are either formed in vivo upon contact with physiological fluids or in vitro by dispersion using hydration media before administration [2]. As shown in Table 1, most studies prepared solid-type proliposomes or freeze-dried liposomes. By using TEM and SEM, most studies provided image proof that proliposomes and freeze-dried liposomes could form multilamellar vesicles upon re-hydration. However, there were not enough data provided regarding the stability of the liposomes in the GI fluids in their studies. Most of the studies did not evaluate the changes of size and zeta potential in SGF and SIF. In addition, there are many cases that did not describe what type of media were used for the measurement of size and zeta potential. It would be more useful to understand the in vivo stability of liposomes if more comprehensive studies had been performed using various media, including water, PBS, SGF, SIF or other simulated GI media. 

#### 3.1.3. Surface Modification

**Figure 5:**
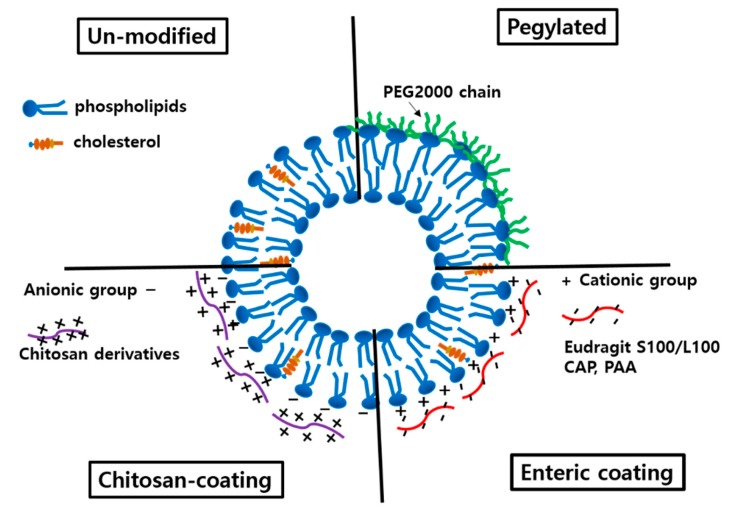
Surface modification of liposomes for in vivo BA enhancement.

As shown in Figure 5, surface modification with polyethylene glycol 2000 or coated with acid-resistant polymers and mucoadhesive chitosan derivatives was applied to protect liposomes from the harsh environment of GI tract [49]. Through enteric coating, more liposomes would survive in the GI tract and hence, prolonged release could be achieved from the small intestine up to the large intestine. In the studies listed in Table 1, enteric coating was accomplished through a charge interaction between positively charged liposomes and negatively charged coating agents such as Eudragit S100 and L100. Enteric coating effect was prominent, showing 3.1- and 5.1-fold increases of BA for docetaxel and sorafenib, respectively, as shown in Table 1 [19,30,63]. In contrast, coating with CAP increased the BA of halofantrine only by 1.4-fold [63]. The difference were the result of the additional components of liposomes, such as bile salt and TPGS, as is the cases for docetaxel and sorafenib. Pegylated liposomes were also tried for daidzein [43]. Pegylated liposomes showed sustained release of daidzein in pH 1.2 and pH 6.9, suggesting prolonged stability in the GI tract. Pegylated liposomes might not be taken up in their intact form by epithelial cells due to steric hinderance by hydrophilic polyethylene glycol chains. Hence, it may be the enhanced stability of the pegylated liposomes in the GI tract that contributes to the improved BA. 

### 3.2. Enhanced Permeability 

**Figure 6:**
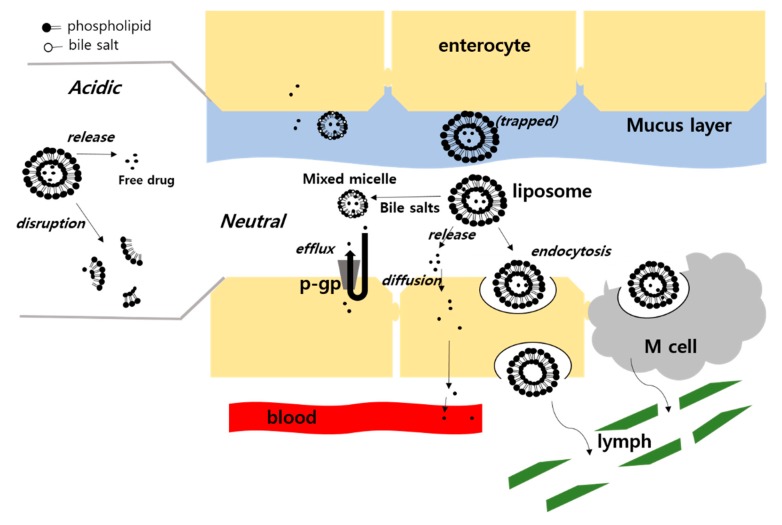
Proposed fate and absorption mechanisms of water-insoluble drugs delivered by liposomes in the GI tract.

In general, conventional liposomes or other nanoparticles have been believed not to cross the mucosal barrier in the GI tract because of their relatively large size. Therefore, it has been suggested that drug is released to be absorbed, or transformed into mixed micelles in the GI tract before being transported to the epithelial cells for absorption, as depicted in Figure 6 [64]. However, M cell-mediated uptake has been also suggested for macromolecular drugs such as insulin, and hence, has drawn attention for the absorption route for liposomes and other nanoparticles [65]. Nevertheless, the absorption of intact liposomes has not been regarded as a significant route because M cells represents only 1% of the total epithelial cell population [66]. There have been various attempts to increase the permeability of the free form of drugs released from liposomes or the encapsulated form in intact liposomes, as described below. 

#### 3.2.1. Cationic Liposomes

There have been attempts to prolong GI residence time and subsequently, increase opportunities for the absorption of drugs using mucoadhesive liposomes. Mucoadhesive property can be obtained through charge interaction with negatively charged mucus. As presented in Table 1, cationic liposomes prepared by adding SA exhibited the highest BA of raloxifen and zaleplon compared to neutral and anionic ones. In contrast, paclitaxel-containing cationic liposomes failed to increase oral BA compared to free drug suspension showing 99% relative BA [45]. However, layer-by-layer coated cationic liposomes with polyelectrolyte remarkably increased the BA of paclitaxel, showing 408% relative BA and suggesting that additional stabilization was needed for in vivo performance to protect inner cationic liposomes. 

#### 3.2.2. Modification with Chitosan and Its Derivatives

Chitosan is a natural cationic polysaccharide and its solubility and functional properties depend on the average molecular weight, the degree of deacetylation and the arrangement of acetamide groups along the main chain [67]. The ability of chitosan to interact electro-statistically with negatively charged residues in mucin glycoproteins is responsible for its mucoadhesive property. In addition, chitosan interacts with a tight junction to facilitate the para-cellular transport of drugs [68,69]. As listed in Table 1, chitosan and its various derivatives such as TMC, CMCS, OACS and GC have been used as coating agents for liposomes. TMC shows good solubility in a broad range of pHs while chitosan is soluble only in acidic media. However, the TMC layer was covered by the CMCS layer because of cell rupture by TMC [38]. The arginine derivative of chitosan, OACS, was also attempted to increase the permeation of the drug in the mucosal barrier [41]. Another chitosan derivative, GC, was newly introduced because it is water-soluble and cationic at physiological pH, while chitosan has a very low solubility above pH 6.5, with the loss of cationic charge [30,70]. Moreover, chitosan coating has been considered to increase the membrane integrity and physical stability of liposomes [71]. 

#### 3.2.3. Incorporation of Bile Salts

Bile salts are endogenous surfactants that play major roles in the absorption of lipids and lipophilic agents. Bile salts can be easily incorporated into liposomal membrane and showed the facilitated absorption of various drugs [72]. Most studies used SDC among various bile salts. As shown in Table 1, SDC-incorporated liposomes increased the BA of curcumin, cyclosporine A, docetaxel, and fenofibrate. In particular, the liposomes consisting of SPC and SDC (3:1) showed comparable BA of cyclosporine A to the commercial microemulsion formulation, showing 120.3% relative BA, while the conventional liposomes without bile salt showed (SPC:CH 5:1) 98.6% relative BA [40]. The authors suggested that the improved BA might have resulted from the absorption of intact vesicles rather than enhanced solubilization because of the limited release of cyclosporine A from the liposomes and microemulsions. The SDC-liposomes and microemulsions released less than 5% in 0.2% sodium lauryl sulfate solution during 12 h at 37 °C. The result show that BA, compared to the commercial microemulsion product, is very encouraging for liposomal oral delivery. 

#### 3.2.4. Modification with Hydrophilic Nonionic Polymers 

In oral delivery using nanoparticles, the mucus layer has been a main hurdle because even very small nanoparticles (6 and 30 nm) can be trapped in the mucus blanket of glycocalyx prior to reaching the apical membrane of epithelial cells [73]. It was disappointing that even mucoadhesive systems resulted in poor particle distribution onto the mucosal surface as well as prompt elimination due to the rapid turnover rate of mucus [74]. Instead of a mucoadhesive system, mucus-penetrating systems have been suggested. Hydrophilic non-ionic polymers such as Pluronic F127 (PF127) and polyethylene glycol can reduce particle adhesion to mucin fibers in the mucus layer, thereby allowing the nanoparticles to diffuse through the low viscosity interstitial fluids between mucin fibers [75]. Pluronic F127-modified liposomes increased the BA of cyclosporine A by 1.3-fold compared to unmodified liposomes, whereas chitosan-modified mucoadhesive liposomes failed to increase the BA [42]. They showed that the mucoadhesive liposomes were trapped in the mucus layer of duodenum and jejunum without reaching ileum 2 h after intragastric administration, while the Pluronic F127-modified liposomes were distributed deeper in mucus layer and reached ileum. 

### 3.3. Enhanced Dissolution 

Several studies incorporated non-ionic surfactants, such as Labrasol, TPGS, and Cremophor EL, as shown in Table 1. In the case of carvedilol, the suggested mechanism for the enhanced BA was improved dissolution rather than active transport of vesicles. In addition, they confirmed that lymphatic delivery was involved in bypassing the pre-systemic metabolism [18]. TPGS was supposed to solubilize curcumin and inhibit the p-gp-mediated efflux pump, although there was no direct evidence provided in the study [37]. Cremophor EL has been used as a solubilizer for various water-insoluble drugs and is known to inhibit the efflux pump [76]. Cremophor EL-incorporated liposomes increased the absorption of cyclosprorin A, which is a water-insoluble p-gp substrate [39]. 

## 4. In Vivo Evidence for Enhanced BA by Liposomes

As shown in Table 1, liposomes have been tested for diverse hydrophobic drugs irrespective of therapeutic category and molecular structure. A distinctive pattern has seldom been found in BA enhancement among drugs, suggesting that liposomal formulation strategies should be applied case by case. In this section, each of the in vivo results for 24 drugs was reviewed in detail. 

### 4.1. Apigenin

Apigenin is a polyphenolic compound and marketed as a dietary and herbal supplement. However, its poor aqueous solubility and rapid metabolism result in low oral BA, which hinders the clinical potential of apigenin. Telange et al. prepared the apigenin-phospholipid complex (APLC) by the incubation of apigenin and phospholipon 90H in a mixture of 1, 4–dioxane and methanol for 2 h at 50 °C, dried, re-dissolved in a mixture of chloroform and methanol, precipitated in hexane, and then vacuum-dried with filtered precipitate [16]. The APLC showed a mean particle size of 107.08 ± 1.30 nm and a zeta potential of −22.35 ± 0.30 mV. According to their solubility study, APLC remarkably increased the solubility of apigenin in water by 37-fold (0.62 ± 0.88 vs 22.80 ± 1.40 µg/mL), while the physical mixture (1:1) of apigenin and phospholipid did so by 10-fold (0.62 ± 0.88 vs 6.13 ± 1.13 µg/mL). They explained that the increased solubility was likely due to the amorphous state of apigenin in APLC based on the differential scanning calorimetry (DSC) and X-ray diffraction (XRD) results. The dissolution of apigenin through the dialysis membrane in phosphate buffered saline (PBS), pH 7.4, containing 1% Tween 20 was higher in APLC compared to the apigenin suspension (51% vs. 28% released after 12 h). The enhanced solubility and dissolution resulted in increased oral BA in rats by 1.5-fold compared to the apigenin suspension.

### 4.2. Carbamazepine

Carbamazepine has been used as one of the first-line antiepileptic drugs and is commercially available as oral-suspension, immediate-release and controlled-release tablets. However, its use has been limited by the high variation in oral bioavailability and erratic behavior due to extensive first-pass metabolism compromising therapeutic effectiveness [77]. Although carbamazepine is well absorbed after oral administration, it has been challenging to formulate because of its lipophilic nature and dissolution rate-limited absorption. There have been several trials to develop improved formulations for oral delivery of carbamazepine, such as solid-dispersion, lipid nanoparticles and the cyclodextrin inclusion complex [17,78]. El-Zein et al. prepared carbamazepine-DMPG 10:1 co-precipitate and showed slightly higher oral BA (1.2-fold) in rabbits compared to the commercially available suspension, Tegretol^®^ [17]. The co-precipitate significantly increased the dissolution rate of carbamazepine in water compared to the free drug suspension, which was likely due to the change of the solid state of carbamazepine into amorphous form in the co-precipitate based on the DSC and XRD results. Although the co-precipitates were not liposomes with a spherical shape, they might have looked like or behaved like liposomes when hydrated in aqueous phase. The authors discussed that it was likely that the bilayer structures were formed and entrapped the solutes during the dissolution process in water and then delivered the drug to the site of absorption. According to the pharmacokinetic results, the co-precipitate exhibited lower variation in AUC (21.6% vs 33.2% of coefficient of variation (CV)) and C_max_ (19.1% vs 63.4% of CV) compared to the suspension, suggesting that the phospholipid-drug co-precipitate could be an improved system to reduce inter-subjects variation.

### 4.3. Carvedilol 

Carvedilol is widely used for the treatment of cardiovascular disease such as hypertension and congestive heart failure. However, its BA is low (25–35%) since it is slightly soluble in water, undergoes extensive first-pass metabolism in the liver, and susceptible to p-gp-mediated efflux [79]. In addition, its solubility is pH-dependent, dissolving in acidic pH and precipitating in basic pH [80]. There has been a report that 78.2–91.8% of carvedilol precipitates under digestion condition [81]. Ghassemi et al. prepared surfactant-enriched liposomes to enhance the oral BA of carvedilol because various surfactants have been reported to possess a p-gp inhibitory effect [18]. The surfactant-enriched liposomes (egg phosphatidylcholine (EPC):cholesterol (CH):surfactant 65:15:20) increased the oral BA of carvedilol compared to the free drug suspension, depending on the type of surfactant in the following descending order: Labrasol^®^ (2.3-fold) > D-α-tocopheryl polyethylene glycol 1000 succinate (TPGS) (2.3-fold) > Tween80 (1.7-fold) > Brij35 (1.4-fold). Non-surfactant liposomes (EPC:CH 85:15) enhanced BA (1.7-fold) as well. According to their additional study, the conventional liposomes demonstrated reduced drug accumulation in Caco-2 cells compared to the free drug suspension. In contrast, the cellular uptake by Labrasol-enriched liposomes was comparable to that by the drug suspension, showing a higher uptake than the conventional liposomes and not affected by ATP depletion with sodium azide. The results of the cellular uptake indicated that the improved BA was likely to be associated with increased dissolution by Labrasol not deteriorating the good permeability property of carvedilol rather than the active transport of Labrasol-enriched liposomes. They also noticed that the increase of BA by drug-containing Labrasol-enriched liposomes was much higher than the concomitant administration of the free drug suspension and empty Labrasol-enriched liposomes. This suggests that the observed enhancement effect by the Labrasol-enriched liposomes was mainly due to the nanostructure in addition to the effects as surfactant and lipid. Moreover, they showed that the BA from the Labrasol-enriched liposomes was reduced by 30% when chylomicron was blocked with cycloheximide pretreatment, indicating the involvement of a lymphatic route which enables drugs to bypass the pre-systemic metabolism. 

### 4.4. Curcumin 

Curcumin is a polyphenolic compound found in *Curcuma longa* and has been known to show a broad range of biological functions, including antioxidant, antimicrobial, anti-inflammatory and anticancer activities [82,83]. However, its clinical use has been limited by the poor oral BA which resulted from low water-solubility, rapid metabolism, hydrolysis in the GI tract and susceptibility to p-gp-mediated efflux [84]. Chen et al. designed mucoadhesive liposomes to enhance the oral BA of curcumin [37]. As a mucoadhesive polymer, N-trimethyl chitosan chloride (TMC), a chitosan derivative, was synthesized because of its good solubility in a wide pH range, unlike chitosan, which is only soluble in acidic media. They prepared mucoadhesive liposomes by coating the liposomes consisting of soybean phosphatidylcholine (SPC), CH, TPGS and curcumin (20:2:12:1) with TMC. The TMC-coating increased mean diameter of the liposomes from 221.4 nm to 657.7 nm and reversed the zeta potential from negative (−9.63 mV) to positive values (+15.64 mV). The uncoated and TMC-coated liposomes greatly increased the oral BA of curcumin by 6.7- and 10.6-fold compared to curcumin suspension. They hypothesized that the improved BA resulted from mucosal adhesiveness by TMC coating and protection of curcumin from the degradation in the GI tract as well as the solubilization of curcumin and p-gp inhibition by TPGS. In addition, it was also discussed that TMC could facilitate paracellular transport by opening a tight junction through ionic interaction with negatively charged cell membrane. 

Unfortunately, TMC was reported to rupture the cells through electrostatic interaction with a negatively charged cell membrane [85]. Tian et al. tried another chitosan derivative, carboxymethyl chitosan (CMCS) instead of TMC as a protective shell for the liposomes [38]. First, liposomes were prepared using SPC and sodium deoxycholate (SDC) (70 mg:25 mg) by the film hydration method, followed by layer-by-layer coating with TMC for the inner layer and then CMCS for the outer layer. TMC-coated and CMCS/TMC-double layer-coated liposomes increased the area under the curve of the plasma concentration-time curve (AUC) of curcumin in rats by 2.3-fold and 5.7-fold compared to the uncoated liposomes. They commented that 6%, 12% and 38% absolute BA were obtained by uncoated, TMC-coated and CMCS/TMC-coated liposomes, respectively, even though pharmacokinetic data after intravenous administration were not presented in the paper. In an organ distribution study performed 24 h after oral administration using in vivo imaging instrument, the CMCS/TMC liposomes exhibited a significantly stronger fluorescence signal of curcumin in the liver, spleen and lung compared to the other two types of liposomes. In contrast, the TMC-liposomes showed very strong signals in the kidney, suggesting that released free from of curcumin was accumulated in the kidney to be excreted. Consistently with the absorption results, the uncoated liposomes demonstrated the negligible fluorescence signals of curcumin in the organs. Based on these results, the authors insisted that CMCS/TMC-liposomes could markedly enhanced paracellular transport by opening a tight junction even though no direct evidence was provided. 

Li et al. prepared flexible liposomes called transferosomes using SPC and SDC (85:15 weight ratio) and then subsequently coated then with silica to protect the liposomes from the harsh environment of the GI tract [36]. The uncoated and silica-coated flexible liposomes enhanced the oral BA in rats by 2.3- and 3.3-fold, respectively, compared to the curcumin suspension. The silica coating reduced the release of curcumin in the artificial gastric and intestinal fluid containing 2% sodium dodecylsulfate (SDS), indicating the protection of a liposomal structure from the GI environment, which consequently might have contributed to the enhanced BA.

As shown in Table 4, pharmacokinetic data show great variations in AUC and C_max_ among studies. The AUC values for curcumin suspension are significantly different between the two research groups, Li et al. and Chen et al., even after being normalized by dose. This difference might have been caused by analytical errors regarding the extremely low BA and plasma concentration. Li et al. used the reverse-phase high-performance liquid chromatography (HPLC) analysis method. However, there was no information on what type of detectors were used. In addition, their plasma concentration curves showed high variation. The other two studies by Chen et al. and Tian et al. measured the plasma concentration of curcumin using HPLC with an ultraviolet/visible (UV/Vis) detector. Recently, Wang et al. reported pharmacokinetic data measured by triple-stage ion trap mass spectrometry coupled with HPLC (HPLC-ITMS/MS/MS) [86]. In the study by Wang et al., curcumin powder suspended in a vehicle consisted of Cremophor, Tween 80, ethanol and water (1:1:1:7 at a volume ratio) showed only 3% BA. We tried to compare the AUCs reported by the three groups and estimate the absolute BA of liposomal formulations using the intravenous AUC value reported by Wang’s group. However, this was not possible due to the remarkable discrepancy in AUC values. Fortunately, Tian’s group reported their own BA values even though the absolute values of AUC and C_max_ were far higher than those reported by Wang’s group, as shown in Table 4. When it was taken into consideration that the vehicle used by Wang et al. contained surfactants and ethanol, which can facilitate oral absorption of drugs, the fact that a BA higher than 6% was achieved by liposomes is very encouraging regarding liposomal oral delivery. Another lesson that we should take from the liposomal curcumin studies is that validation for analytical methods should be thoroughly performed and more sensitive analytical tools applied to establish the reliability of pharmacokinetic data when measuring the plasma concentration of drugs with low BA.

### 4.5. Cyclosporine A

Cyclosporin A is a hydrophobic cyclic peptide widely used as immunosuppressant. Its oral BA is very low because of its water-insoluble property, its first-pass metabolism by CYP 3A4 and susceptibility to p-gp-mediated efflux [87]. 

Shah et al. prepared proliposomes consisting of cyclosporine A, EPC and Cremophor EL (1:10:0.5) using directly compressible lactose as the carrier [39]. The encapsulation efficiency and mean particle size of hydrated proliposomes were 98.4% and 12.39 µm, respectively. The proliposomes showed a 9.6-fold increase of oral BA of cyclosporine A compared to the free drug suspension in rats. In addition, the proliposomes exhibited a BA comparable to the commercial microemulsion preparation. Although there was no further study regarding the absorption mechanism, it appeared that the improved BA was due to the solubilization of cyclosporine A by phospholipid and the surfactant, Cremophor EL. 

Guan et al. evaluated liposomes containing bile salt sodium deoxycholate (SDC) [40]. The lipsosomes consisting of SPC/SDC (3:1) or SPC/CH (5:1) showed similar C_max_ and AUC to the commercial microemulsion, Sandimmun Neoral^®®^, showing 120.3% and 98.6% relative BA, respectively, which did not appear to be statistically significant. The liposomes and microemulsion released less than 5% drug for 12 h when measured by the dynamic dialysis method, which implied that the enhanced oral BA was probably due to the facilitated permeation by bile salt or liposomes rather than improved release. 

Chen et al. modified the liposomes with chitosan (CS-Lip) or Pluronic F127 (PF127-Lip) to prepare mucoadhesive or mucus-penetrating liposomes, respectively [42]. The un-modified liposomes (Lip) consisted of EPC, CH and cyclosporin A (28:5:1). The PF127-Lip increased C_max_ and AUC_0–t_ by 1.73- and 1.84-fold, respectively, compared to the un-modified liposomes, while chitosan-modified ones decreased both parameters. According to their stability study in simulated gastric fluid (SGF) and simulated intestinal fluid (SIF), the positively charged CS-Lip aggregated in SIF to be trapped by mucus and remained mainly in the upper region of the GI tract, resulting in limited penetration. On the contrary, PF127-Lip remained stable in both SGF and SIF and was found in the whole region of the GI tract, showing a mucus-penetrating ability. 

Another study adopted *N*-octyl-*N*-arginine-chitosan (OACS), a derivative of chitosan, to evaluate permeability-enhancing ability by arginine-rich peptide and drug-loading capacity by the amphiphilic polymers to modify the liposomes consisted with SPC and CH (20:1) [41]. The OACS-modified liposomes (using OACS with 10% of octyl substitution and 10% of arginine substitution) increased oral BA of cyclosporine A by 3.2-fold compared to the free drug suspension, while unmodified liposomes did so by 1.7-fold. In addition, OACS-modified liposomes showed a 1.5-fold higher oral BA compared to the commercially available microemulsion. OACS-modification slowed down the release rate of the drug from the liposomes. Moreover, OACS-liposomes presented the highest absorption of drug at jejunum in an in situ single pass perfusion study. 

There are significant differences between AUC and C_max_ among the studies, even though the parameters were normalized by the dose shown in Table 5. The difference in particles size of the liposomes might have caused this discrepancy. The liposomes tested by Shah et al. showed an approximately 100-fold larger particle size compared to those tested by Chen et al. and presented the lowest AUC among the studies listed in Table 5. Overall, smaller liposomes provided higher AUC even though there could be other factors such as liposomal composition and surface modification. 

### 4.6. Daidzein 

Daidzein, a natural compound easily found in soybeans and a number of plants, has been reported for its pharmacological activity in the prevention and therapy of cardiovascular disease, several types of cancer, osteoporosis and menopausal symptoms [88]. Due to its low water solubility and first-pass metabolism, daidzein shows poor oral BA [89]. Wang et al. tested long-circulating nanoliposomes with SPC, CH and DSPEPEG2000 (55:40:5) to increase the oral BA of daidzein [43]. The long-circulating liposomes exhibited a sustained release of daidzein in pH 1.2 and pH 6.8 media, which resulted in delayed T_max_ and similar C_max_ compared to the free drug suspension with a 2.5-fold increase of AUC_0–∞_. In general, long-circulating liposomes modified with polyethylene glycol (PEG) chains have been used for parenteral formulation to stabilize liposomes in blood circulation and reduce undesirable uptake by the reticuloendothelial system [90]. Through the case of daidzein oral delivery using pegylated liposomes, the PEG moiety was proven to be effective in stabilizing liposomes even in the GI tract. 

### 4.7. Docetaxel 

Docetaxel is a widely used anticancer agent and available only for intravenous administration due to its extremely low oral bioavailability (approximately 10%) which results from its poor aqueous solubility, high susceptibility to p-glycoprotein-mediated efflux and first-pass metabolism [3,91]. Kim et al. studied freeze-dried Eudragit-coated liposomes and showed a 3-fold increase in bioavailability compared to free DTX solubilized in a mixture of polysorbate 80, ethanol and saline (20:13:67) [19]. The liposomes consisted of EPC, CH, stearylamine (SA) and SDC (39:7.8:0.018 mmol), and presented a cationic surface charge because of SA. The cationic charge enabled the coating of liposomes with Eudragit L100 and S100 (4:1) through electrostatic interaction. It is likely that the Eudragit-coating protected the liposomes in acidic environment, leading to prolonged docetaxel release in the intestine. In addition, they tried to tackle the stability issue of liposomes by freeze-drying the Eudragit-coated liposomes in the presence of 20% trehalose and 10% mannitol. The mean particle size of liposomes increased by Eudragit-coating and the freeze-drying process, which was not considered to be significant because the mean particle size still remained in the sub-micron range (116.4 ± 5.9 nm and 204.9 ± 36.8 nm, respectively). Zeta potentials were reversed by Eudragit-coating (−31.1 ± 0.6 mV from +19.3 ± 1.6 mV) and not significantly changed by freeze-drying (−22.9 ± 4.0 mV). When administered to rats, free docetaxel showed 1.91% absolute bioavailability, while docetaxel-loaded Eudragit-coated liposomes showed 5.92%. It is likely that the prolonged release in the small intestine up to the colon contributed to the enhanced bioavailability of Eudragit-coated liposomes. The authors did not show any further mechanistic studies. Their liposomes contained only 0.106% drug compared to the polymeric components in the liposomes and hence, the greatly high amount of phospholipids might have improved the intestinal permeability or protected the docetaxel from p-gp-mediated efflux. 

### 4.8. Dronedarone

Dronedarone, an antiarrhythmic agent, has a low oral BA of approximately 4% without food and 15% with a high-fat meal [92]. The low BA of dronedarone has been ascribed to the poor solubility and extensive first-pass metabolism. Kovvasu et al. prepared proliposomes using dimyristoyl-phosphatidylglycerol sodium (DMPG Na) and CH (1:2) with microcrystalline cellulose (MCC) as the carrier [20]. They administered it via oral gavage after suspending both free dronedarone HCl and proliposomes in 5% hydroxypropylmethylcellulose (HPMC) solution. The proliposomes showed a 1.5-fold increase of oral BA of dronedarone compared to the free drug suspension. The authors also exhibited that the proliposomes increased apparent drug permeability through Caco-2 cells by 2.5-fold. In addition, the physical form of the drug was changed from crystalline to amorphous form according to the DSC study. 

### 4.9. Fenofibrate 

Fenofibrate is a lipophilic drug used to treat abnormal lipid levels. However, its therapeutic efficacy has been compromised due to its insolubility in water [93]. Chen et al. prepared liposomes with a combination of SPC and SDC (4:1) and showed a 5.3-fold increase of BA compared to the micronized fenofibrate in dogs, while non-bile salt containing conventional liposomes composed of SPC and CH (4:1) exhibited 3.2-fold increase [21]. The authors suggested that the superior BA by SPC/SDC liposomes might be due to the facilitated transition from the vesicle structure to mixed micelle by the bile salt. 

### 4.10. Flutamide

Flutamide is a nonsteroidal antiandrogen that blocks the action of both endogenous and exogenous testosterone by binding to the androgen receptor and is used to treat androgen-sensitive prostate carcinoma. It has low oral BA due to its low aqueous solubility and extensive first-pass metabolism [94]. Youssef et al. formulated flutamide-containing polymerosomes using amphiphilic copolymer polyethylneglycol-polycaprolactone (PEG-PCL) as stable and sustained release formulation [22]. The polymerosomes increased oral BA by 2.6-fold compared to flutamide suspension. They also investigated liposomal formulation consisting of SPC and CH and showed no significant increase of oral BA of flutamide compared to the drug suspension. The polymerosomes were stable during storage, showing no significant changes in particle size, polydispersity index and zeta potential for 5 months while liposomes were not stable. In simulated intestinal fluid, the polymerosomes showed a much slower release of flutamide than the liposomes, while both polymerosomes and liposomes were stable in simulated gastric fluid, releasing less than 20% flutamide adsorbed on the surface of the vesicles. In addition, the polymerosomes remained stable in bile salt-containing media, showing no turbidity changes. They suggested that the enhanced oral BA by polymerosomes was attributed to the high stability in the GI tract and sustained release. In some other studies for lovastatin and vinpocetin, SPC/CH proliposomes increased BA. The reasons that flutamide liposomes were not effective in BA enhancement might be not only the instability of liposomes prepared in liquid form, but also the absence of additional effects by silicified MCC and sorbitol contained in the proliposomes of lovastatin and vinpocetin. There have been reports showing that pharmaceutical excipients such as cellulose derivatives and sorbitol can change the dissolution behavior and bioavailability of drugs on a case-by-case basis [95,96]. Based on these possibilities, investigate the effects of carriers used for proliposomes on the behavior of liposomes and drugs in the GI tract need to be investigated. 

### 4.11. Halofantrine

Halofantrine is a drug used to treat malaria and its absorption is incomplete and erratic because of its high lipophilicity [97]. In rats, absolute oral BA of halofantrine was 20–30% in the fasted state and increased with high-fat foods [63]. Brocks and Betageri prepared proliposomes with halofantrine and DSPC (1:3) followed by enteric-coating with cellulose acetate phthalate (CAP) using a spray dryer [23]. The enteric-coated proliposomes increased the BA of halofantrine by 1.4-fold compared to the free drug suspension in rats. Although they did not perform any further mechanistic study, they suggested that BA enhancement might be mediated by the solubilization of halofantrine by phospholipid. 

### 4.12. Indomethacin 

Indomethacin is a non-steroidal anti-inflammatory drug (NSAID) that belongs to BCS class II drug. Sugihara et al. tried to improve the oral BA of indomethacin by using liposomes modified with mucoadhesive polymer, chitosan [24]. They also controlled the particle size of chitosan-coated liposomes to fall within the submicron range (270–310 nm). In fasted rats, the absolute BA of indomethacin was 92.9% and 93.1% for the uncoated and chitosan-coated liposomes, respectively. In contrast, free drug solution and suspension showed 50.5% and 37.1% of oral BA, respectively. In addition, the chitosan-coated liposomes showed a still higher BA (75.2%) even with meals, suggesting no loss of carrier function which may have been caused by the destruction of the liposomes in the presence of foods. According to the retention profiles in the GI tract segments, the chitosan-coated liposomes remained longer in all the segments including stomach, duodenum, jejunum and ileum than the uncoated ones indicating the contribution of mucoadhesive property to the enhanced BA. 

### 4.13. Isradipine

Isradipine, a calcium antagonist, has low oral BA due to its poor water-solubility and extensive first-pass metabolism [98]. Bobbala et al. showed a 2.0-fold increase of oral BA in rats with proliposomes consisted with hydrogenated soybean phosphatidylcholine (HSPC) and CH (1:1) compared to the isradipine suspension in 0.5% sodium CMC [25]. They also exhibited transformation of drug crystal into amorphous state through DSC, scanning electron microscopy (SEM), powder XRD (PXRD) and Fourier-transform infrared spectroscopy (FT-IR) studies, which resulted in a much higher dissolution of the drug from the proliposomes than drug powder in PBS, pH 7.4. In addition, the proliposomes increased the permeability of isradipine across rat intestine by 2.4-fold compared to the pure drug suspension. The authors postulated that the drug was absorbed as encapsulated in the intact liposomes and the higher absorption might result from the delivery of the drug by vesicular endocytosis. In addition, they assumed that the first-pass metabolism of isradipine might be avoided through increased transport of liposome to the lymphatic system. 

### 4.14. Lopinavir 

Lopinavir, an antiviral agent, is classified as BCS class II or IV and susceptible to p-gp and pre-systemic metabolism by CYP450 3A4 [99]. Hence, administered alone, lopinavir shows insufficient bioavailability. However, its bioavailability is greatly increased by a low dose of ritonavir, a potent inhibitor of CYP450 3A4 and p-gp. Even though lopinavir is marketed as a fixed-dose combination tablet with sub-therapeutic dose of ritonavir, there has been a need for ritonavir-free formulation because ritonavir has adverse effects such as glucose intolerance, gastrointestinal intolerance, lipid elevation and perioral paresthesia. Patel et al. developed pro-liposomal formulations for lopinavir based on the quality-by-design approach [44]. Their optimized pro-liposomes for lopinavir consisted of 3.75 µmol of lipid mixture (HSPC:CH 7:3), lopinavir at a ratio of lipid:drug 8.9:1 and 2250 mg of spray-dried mannitol. The mean particle size and zeta potential were 659.7 ± 23.1 nm and −24.8 ± 0.21 mV, respectively. In rats, the proliposomal formulation improved oral the bioavailability of lopinavir by 2.2-fold compared to free drug suspension in 0.5% sodium carboxymethylcellulose. The proliposomes also showed comparable BA (1.16-fold increase) to the commercial product (a fixed dose combination of 500 mg lopinavir and 50 mg ritonavir). The proliposomes exhibited a much higher 60-min dissolution in pH 6.8 media compared to lopinavir suspension (95% vs. 55%). The researchers also proved that the physical state of lopinavir was transformed into amorphous form in proliposomes using DSC, XRD, FT-IR and SEM. Based on these results, the enhanced absorption of lopinavir in proliposomes was likely due to the improved dissolution. In addition to the enhanced dissolution, Patel et al. also showed improved ex-vivo permeability of proliposomes through rat intestine (1.99-fold compared to the lopinavir suspension) and suggested that it might have resulted from the uptake of intact liposomes through endocytosis, the hindered barrier function through phospholipid-induced cellular membrane fluidization or p-gp inhibition by excipients in the proliposomes. In addition, the authors discussed that the improvement of absorption could be explained by the relatively small size of liposomes, similarity between lipid bilayers and biomembranes, better adherence to biomembranes and formation of mixed micelles with bile salts secreted in the intestine. Mixed micelle would solubilize and transport the drug into the lymphatic system, bypassing pre-systemic metabolism. However, they did not provide any experimental evidence for this hypothesis. 

### 4.15. Lovastatin 

Lovastatin has a low solubility and a high permeability with extensive first-pass metabolism [100]. Lovastatin undergoes hydroxylation from its inactive lactone to active metabolite (lovastatin acid) in the liver. Yanamandra et al. tried to decrease the hepatic first-pass metabolism of lovastatin by orally delivering using proliposomes [26]. They selected a proliposomal formulation showing the highest dissolution rate (SPC:CH:drug 0.45:0.05:1) for the in vivo absorption study in rats. The proliposomal formulation increased the BA of the parent drug by 1.6-fold compared to the free drug suspension while decreased metabolite by 50% (121.54 vs 252.49 of metabolite AUC). They hypothesized that the first pass metabolism might have been avoided by the slower release of lovastatin from the proliposomal formulation. Even though this hypothesis warrants further studies, it was clear that the proliposomes could improve dissolution of lovastatin, resulting in increased BA. 

### 4.16. Nisoldipine

Nisoldipine is a calcium channel blocker and its absolute BA reported was approximately 5% with inter-subject variations [101]. The poor BA of nisoldipine has been regarded due to poor aqueous solubility, extensive pre-systemic metabolism and susceptibility to p-gp-mediated efflux. The pre-systemic metabolism of nisoldipine decreases from the proximal to the distal parts of the intestine. It is commercially available as a film-coated extended release tablet. Nekkanti et al. tried proliposomes and self-microemulsifying drug delivery system (SMEDDS) platforms to improve the oral BA of nisoldipine [27]. The proliposomes and SMEDDS increased the oral BA of nisoldipine by 3.0- and 2.4-fold, respectively, compared to drug suspension in rats. In addition, they showed increased dissolution of nisoldipine in water from the proliposomal powder filled in hard gelatin capsules and enhanced transport through parallel artificial membrane permeability assay (PAMPA) and everted rat intestine compared to nisoldipine suspension. However, it was not clear whether liposomal structure was a major contributor for the enhanced transport rather than increased dissolution because Nekkanti et al. compared in vitro permeation with nisoldipine suspension, not with nisoldipine solution. 

### 4.17. Paclitaxel 

Paclitaxel (PTX) has been a great challenge in the pharmaceutical field because of its notoriously poor aqueous solubility and p-gp-mediated efflux in spite of its broad spectrum of anticancer efficacy. It has been only available in injectable dosage form due to the low oral BA (2% of absolute BA in rats) and there have been extensive attempts to develop oral dosage forms for more than three decades [1,102]. Finally, a novel oral formulation, DHP107, was approved in South Korea in 2016, which is an oily liquid composed of monoolein, tricaprylin, polysorbate 80 and 1% PTX and supposed to be mucoadhesive in the GI tract [103,104]. This success has casted a light upon the pharmaceutical research and soon there, would be more oral dosage forms of paclitaxel that can reach clinical application. 

Jain et al. prepared polyelectrolyte-stabilized multilayered liposomes by layer-by-layer coating methods as an oral delivery system for PTX [45]. Anionic polyelectrolyte, polyacrylic acid (PAA), was layered on to the cationic liposomes consisting of SPC, CH, SA and PTX in the first step and then, the single layer coated liposomes were coated with a cationic polyelctrolyte, polyallylamine hydrochloride (PAH). In addition, the liposomes were lyophilized with mannitol for stability during storage. The double-layer-coated cationic liposomes remained stable in the SGF, pH 1.2 and SIF, pH 6.8 without significant changes in encapsulation efficiency and other physicochemical properties, such as mean particle size, polydispersity index and zeta potential, while the uncoated cationic and one-layer-coated anionic liposomes were unstable, showing more than 50% reduction of encapsulation efficiency. The double layer-coated liposomes (PAH-PAA-PTX-layersomes) showed 4-fold higher AUC compared to the PTX suspension in rats, while the uncoated (PTX-liposomes) and single-layer coated liposomes (PAA-PTX-liposomes) showed only 1.0- and 1.3-fold increase, respectively. However, the improved oral BA is not likely to be sufficient yet compared to the plasma concentration by intravenous formulations. The absolute BA can be roughly estimated by referring yo the AUC reported by Li et al. [105]. In the study by Li et al., the mean AUC was 17.65 µg·h/mL after intravenous administration of Taxol^®®^ formulation at a dose of 4 mg/kg into rats. The mean AUCs reported by Jain et al. were 3127.7 ng·h/mL and 767.15 ng·h/mL for the PAH-PAA-PTX-layersomes and free drug suspension, respectively when administered orally at a dose of 5 mg/kg as PTX. Based on these AUCs, the estimated absolute BA would be 14.2% for the PAA-PTX-liposomes, while the free drug suspension 3.4%. The approved oral formulation, DHP107, showed approximately 23% absolute BA at a dose of 50 mg/kg in mice and showed comparable antitumor effects to the intravenous Taxol^®®^ (10 mg/kg) [106]. 

### 4.18. Piroxicam 

Piroxicam, one of the widely used NSAIDs, is BCS class II drug. Mirza et al. prepared solid dispersion of piroxiam using various phospholipids such as dimyristoylphosphatidylglycerol (DMPG), dimyristoylphosphatidylcholine (DMPC), dipalmitoylphosphatidylcholine (DPPC), and distearoylphosphatidylcholine (DSPC) [28]. Even though their preparation was referred to as solid dispersion, the preparation was included in this review as liposomal formulation because it is likely that the prepared drug-phosphlipids mixture would form liposomal-like structure when hydrated with intestinal aqueous media. They performed an in vivo absorption study in rats using DMPG-piroxicam (15:1 *w*/*w*) solid dispersion which showed the highest dissolution of the drug among various compositions. Unfortunately, it did not increase the oral BA of piroxicam, showing insignificant difference of AUC (1210 ± 254 vs. 921 ± 207 µg·h/mL) from the piroxicam suspension, even though time to maximum plasma concentration (T_max_) was shortened significantly from 5.5 to 2.0 h by solid dispersion. 

### 4.19. Raloxifen

Raloxifen, a selective estrogen receptor modulator, has been used to prevent osteoporosis in postmenopausal women and showed only 2% oral BA due to extensive first-pass metabolism as well as poor water solubility [107]. Similarly to the case of zaleplon in variously charged proliposomes, enhanced oral BA of raloxifen was observed in rats when delivered in neutral, anionic and cationic proliposomes by 2.4-, 2.6- and 3.4-fold, respectively, compared to free drug suspension, which could be ascribed to the increased dissolution of amorphous form and permeability through the intestine [29]. 

### 4.20. Sorafenib

Sorafenib is a kinase inhibitor approved for the treatment of advanced renal cell carcinoma and has drawn attention as a good candidate for colorectal cancer. However, its clinical use has been hampered by low oral BA and adverse events such as diarrhea and GI bleeding [108,109]. A commercially available tablet contains sorafenib tosylate and shows 38–49% of relative BA compared to oral solution due to the poor water solubility of sorafenib tosylate [110]. In addition, large interpatient variations have been reported in the pharmacokinetics of sorafenib, which was supposed to result from slow dissolution in the GI tract [111]. Zhao et al. tried to develop an improved delivery system to prolong the residence time in the colon to increase drug absorption using enteric-coated liposomes [30]. They prepared negatively charged liposomes composed with DPPC, DPPG, TPGS, CH and drug (8:1:2:4:1). The negatively charged liposomes were subjected to the first coating with positively charged glycol chitosan (GC) and then to the second layer enteric coating with Eudragit S100. The coated liposomes were obtained as pellet by centrifugation after coating. The enteric coated liposomes showed a 5.1-fold increase of oral BA compared to the free drug, while the uncoated and single-layer coated ones showed 2.9- and 3.0-fold increase, respectively. However, it was not clearly mentioned that the free drug control for the absorption study was a solution or suspension, which makes us unable to compare its relative bioavailability with the commercially available tablet formulations. Considering the solubility of sorafenib tosylate in water (0.00171 mg/mL predicted with ALOGPS), the control appeared to be a suspension rather than a solution. In addition to the increased AUC, the enteric coated liposomes showed delayed T_max_, which is likely to be explained by the better stability (higher percentage of remaining drug) of the coated liposomes in pH 1.2 media than the uncoated ones. 

### 4.21. Silymarin and Dehydrosilymarin 

Silymarin, extract of milk thistle seeds, has been widely used in various liver disorders mainly because of its antioxidant and anti-inflammatory activity [112,113]. Silymarin is classified as BCS class II or IV agent with poor water-solublility and low intestinal permeability [114]. Consequently, silymarin is poorly absorbed (20–50%) from the gastrointestinal tract and has a low oral BA from oral formualtions [115]. Xiao et al. tried to increase oral BA of silymarin using proliposomes consisting of phospholipid (approximately 82% phosphatidylcholine) and mannitol [31]. According to their study, the proliposomal powder showed 3.4-fold increase of oral BA compared to silymarin powder in beagle dogs. The proliposomes exhibited almost complete dissolution in 20 min in pH 1.2 and pH 6.8 media, while free drug power showed negligible dissolution in 90 min. 

Similar to silymarin, dehydrosilymarin, a derivative of silymarin, shows low oral BA due to its poor water-solubility [113]. Chu et al. prepared proliposomes using soybean phospholipids, CH, isopropylmyristate (IPM), sodium cholate and mannitol and showed a 2.2-fold increase of the oral BA of dehydrosilymarin in rabbit compared to the free drug suspension [32]. They also showed a correlation between the improved BA and the enhanced release of drug from the proliposomes in pH 1.2 and pH 6.8 dissolution media containing 0.1% SDS. They suggested that the enhanced BA by the proliposomes could be explained by the small particle size, the presence of bile salts and similarity between the liposomal bilayer and biomembranes. 

### 4.22. Tacrolimus 

Tacrolimus is a potent immunosuppressant and exhibits low oral BA due to its poor aqueous solubility, p-gp-mediated efflux and extensive pre-systemic metabolism [116]. A commercial product exhibits approximately 25% of BA with large inter-subjects variation. Nekkanti et al. prepared proliposomes composed of tacrolimus, DSPC and CH (1:2:0.5) with a mean particle size of 858 nm [33]. Compared to free drug suspension, the proliposomes exhibited a 1.9-fold increase of AUC, which is consistent with the enhanced permeation through PAMPA and everted rat intestine. The enhanced BA was likely due to the increased dissolution that resulted from the transformation of crystalline tacrolimus to amorphous state in the liposomes.

### 4.23. Vinpocetin

Vinpocetine is a derivative of vincamine and used in the prevention and treatment of ischaemic stroke and other cerebrovascular diseases [117]. Vinpocetine has a poor water-solubility and remarkable first pass metabolism, which results in low oral BA (7% in human) [34]. In addition, dissolution of vinpocetine was much higher in low pH media, while it was very poor in the intestinal tract [118]. Xu et al. prepared proliposomes with SPC, CH and sorbitol and showed a 3.5-fold increase of oral BA compared to drug suspension in rabbits [34]. Similarly to other studies, drug powder showed complete 30-min dissolution in pH 1.2 media and negligible dissolution in pH 6.8 media. On the contrary, the proliposmes showed almost complete 30-min dissolution in both media, pH 1.2 and pH 6.8. According to the concentration–time profile, the proliposomes showed the first peak at 1 h and the second at 3 h after administration, while the free drug suspension showed no second peak. To elucidate the second peak, they administered the proliposomes after filtration to remove un-encapsulated vinpocetin. The filtered proliposomes showed a significantly lower first peak compared to the un-filtered proliposomes. The authors believe that the first peak was mainly due to the absorption of the un-encapsulated free drug while the second peak was due to the absorption of the intact liposomes. 

### 4.24. Zaleplon

Zaleplon is a hypnotic drug used in insomnia which undergoes extensive hepatic metabolism, resulting in approximately 30% oral BA [119]. It also shows dissolution rate-limited absorption due to its poor solubility in water. Janga et al. compared oral BA of zaleplon encapsulated in neutral, anionic and cationic proliposomes which were prepared using HSPC, CH (1:1) and mannitol for neutral liposomes and by adding dicetylphosphate (DCP) and SA (10 mol% of total lipid) to the neutral proliposomal formulation for anionic and cationic liposomes, respectively [35]. The cationic proliposomes increased the oral BA of zaleplon by 4.6-fold compared to the free drug suspension, and neutral and anionic ones by 2.0- and 3.0-fold, respectively. The reason for the highest oral BA by cationic liposomes is likely the electrostatic attraction to negatively charged proteins in the outer membrane of the intestinal epithelial cells. The dissolution of zaleplon was not significantly different among various charged proliposomes, while all the proliposomes showed higher dissolution compared to the free drug suspension control. Their DSC and PXRD studies demonstrated transformation of the crystalline state of zaleplon to amorphous state. Moreover, an in situ perfusion study exhibited the increase of the permeability coefficient and the absorption rate constant in the proliposomal formulations in the following descending order: cationic > anionic > neutral > control. 

## 5. Future Trends and Missions

### 5.1. Ligand Modification for Active Absorption

Referring the fact that various nutrients are absorbed through the transcellular route using receptor-mediated endocytosis [101,120], liposomes modified with ligands can be utilized to enhance the absorption of poorly permeable drugs. There have been several studies showing significant absorption of hydrophilic drugs such as vancomycin and insulin using liposomes modified with fatty acids, biotin, lectins and mannose [121]. In spite of its high efficacy, receptor-mediated endocytosis may not be a single mechanism for the enhanced oral absorption of ligand-modified liposomes. The enhanced oral BA may be attributed to accumulation at the site of absorption and sustained release of the encapsulated drugs by ligand–receptor interaction as well. The absorption of most water-insoluble drugs is dissolution-limited rather than permeability-limited. Hence, increased accumulation at the site of absorption by ligand modification will be a strategy worth trying to improve the absorption of water-insoluble drugs even though the small population of M cells present in the gut and receptor-mediated endocytosis is not sufficient. 

### 5.2. Circumvention of Efflux Pump

A membrane-associated drug efflux transporter, p-gp, is expressed in the apical membrane of enterocytes in the small intestine and plays a key role in reducing the systemic absorption of various substrates. Various water-insoluble drugs suffer from the poor permeability mediated by efflux as well as dissolution-limited absorption [122]. In addition, commonly used pharmaceutical excipients and synthetic phospholipids have been known to show p-gp inhibitory activity, which include TPGS, Tween 80, Tween 20, cetrimonium bromide, Cremophor EL, Solutol HS, sodium carbocymethylcellulose, Brij58 and phospholipids such as 1,2-dioctanoyl-*sn*-glycero-3-phosphocholine (8:0 PC) and 1,2-didecanoyl-sn-glycero-3-phosphocholine (10:0 PC) [123,124]. As shown in Table 1, some studies incorporated p-gp inhibitor into liposomal composition and the enhanced BA was likely partly mediated by p-gp inhibition, although they did not show any direct evidence regarding p-gp inhibition in vivo. The above-mentioned p-gp inhibitors are usually types of surfactants and phospholipids and hence, can be easily incorporated into liposomal membrane and show additional or synergistic effects on solubilizing activity.

### 5.3. Identification of Absorption Mechanisms 

The mechanisms of oral delivery of liposomes have not been fully understood yet, despite the significant number of in vitro studies on the dissolution and permeation enhancement [8]. According to a meta-analysis performed in 2015, liposomes clearly improved solubility and permeability, showing a 127.4% and 59.5% increase, respectively [10]. Water-insoluble drugs can be solubilized into sub-micron sized liposomes, subsequently being released at a sustained mode in the GI tract or transferred into mixed micelles formed in the presence of bile salts, as suggested in Figure 6. Two absorption pathways were inferred: endocytic uptake of intact liposomes or passive diffusion of released free drug. The contribution of M cells should be identified as well in terms of endocytic uptake. In addition, it should be determined whether liposomes or cargos can direct either blood circulation or lymphatics after permeation through the epithelial barrier. Most in vivo studies only showed enhanced BA through a pharmacokinetic analysis in animals lacking comprehensive mechanistic investigations. In recent years, techniques regarding the GI digestion fate of liposomes and in vivo imaging have been suggested and they will be useful to identify the mechanism [125]. 

## 6. Conclusions

There is a significant amount of in vivo evidence that liposomes can increase the BA of various water-insoluble drugs through solubilization combined with permeation enhancement and sustained release in the GI tract. For the stability during storage and in the GI tract, solid proliposomes and freeze-dried liposome were introduced in which drugs present as amorphous state rather than crystalline form to increase the dissolution rate. Enteric coating was applied to protect the liposomes from premature disruption in the harsh gastric environment. In addition, mucoadhesive polymers, mucus-penetrating polymers and bile salts were also incorporated to enhance the permeability of liposomal drugs. However, more comprehensive mechanism studies are warranted to understand the in vivo fate of liposomal drugs. Despite some doubts, in vivo evidence has indicated that liposomes can be promising carriers for oral delivery, possessing easily modifiable properties.

## Figures and Tables

**Table 1:** Characteristics and in vivo bioavailability of liposomal formulations for various water-insoluble drugs.

Drugs (Therapeutic Category)	Liposome Composition	Encapsulation Efficiency (%)	Physical Forms	Study Subject	Relative BA (fold)	Comparator	Reference
BCS Class II drugs							
Apigenin (herbal supplement)	Phospholipid 90H	93.3%	Solid: proliposome (mannitol)	Rat	1.5	Free drug suspension	[16]
Carbamazepine (antiepilectic)	Drug:DMPG (1:1)	ND	Solid: co-precipitate	Rabbitt	1.2 (NS)	Tegretol suspenstion	[17]
Carvedilol (cardiovascular)	EPC:CH:Labrasol (65:15:20)	79.8%	Liquid: liposome dispersion	Rat	2.3	Free drug suspension	[18]
Docetaxel (anticancer)	EPC:SA (1:0.2) with SDC and coating with Eudragit L100/S100 (4:1)	33.6%	Solid: Freeze-dried liposomes (trehalose, mannitol)	Rat	3.1	Free drug solution in polysorbate 80/ethanol/saline (20:13:67)	[19]
Dronedarone (antiarrhythmic)	DMPG Na:CH (1:2)	84%	Solid: proliposomes (MCC)	Rat	1.5	Free drug suspension	[20]
Fenofibrate (antilipidemic)	SPC:SDC (4:1)	88.2%	Liquid: liposomal dispersion	Dog	5.1	Micronized fenofibrate in capsule	[21]
Flutamide (antiandrogen)	SPC:CH (4:1 *w*/*w*)	70.6%	Liquid: liposomal dispersion	Rat	0.9	Free drug suspension	[22]
Halofantrine (antimalarial)	DSPC:Drug (3:1) Coating with CAP	ND	Solid: proliposomes (enteric coating)	Rat	1.4	Free drug suspension	[23]
Indomethacin (NSAID)	DSPC:DCP:CH (8:2:1) coating with chitosan	ND	Liquid: liposomal dispersion	Rat	1.8	Free drug solution	[24]
Isradipine (calcium antagonist)	HSPC:CH (1:1)	96.8%	Solid: proliposomes (mannitol)	Rat	2.0	Free drug suspension	[25]
Lovastatin (antilipidemic)	SPC:CH (9:1)	85.8%	Solid: proliposomes (silicified MCC)	Rat	1.6	Free drug suspension	[26]
Nisoldipine (calcium channel blocker)	DMPC:CH (4:1)	85.6%	Solid: proliposome (MCC)	Rat	3.0	Free drug suspension	[27]
Piroxicam (NSAID)	DMPG	ND	Solid: solid dispersion	Rat	1.3 (NS)	Free drug suspension	[28]
Raloxifen (estrogen receptor modulator)	HSPC:CH with DCP or SA	94.2% (cationic) 93.2% (anionic) 93.9% (neutral)	Solid: proliposomes (mannitol)	Rat	3.4 (cationic); 2.6 (anionic); 2.4 (neutral)	Free drug suspension (processed without lipids)	[29]
Sorafenib tosylate (anticancer)	DPPC:DPPG:TPGS:CH (8:1:2:4) Coating with Glycol chitosan & Eudragit S100	94.6% (uncoated) 96.6% (glycol chitosan-coated) 89.7% (double layer coated)	Liquid: liposome dispersion	Rat	2.9 (uncoated); 3.0 (glycol chitosan-coated); 5.1 (EudragitS100/glycol chitosan coated)	Free drug	[30]
Silymarin (hepatoprotective)	Phospholipid (82% PC)	92.6%	Solid: proliposomes (mannitol)	Dog	3.4	Powder	[31]
Dehydrosilymarin (hepatoprotective)	SPC 0.3 g CH 0.075 g IPM 0.2 g Sodium cholate 0.2 g	70–80%	Solid: proliposomes (mannitol)	Rabbit	2.2	Free drug suspension	[32]
Tacrolimus (immunosuppressant)	DSPC:CH (4:1)	approx. 70–80%	Solid: proliposomes	Rat	1.9	Free drug suspension	[33]
Vinpocetine (Cardiovascular)	SPC:CH (9:1, *w*/*w*)	86.3%	Solid: proliposomes (sorbitol)	Rabbit	3.5	Free drug suspension	[34]
Zaleplon (hypnotic)	HSPC:CH (1:1) with DCP or SA	93.8% (cationic) 92.5% (anionic) 94.6% (neutral)	Solid: proliposomes (mannitol)	Rat	4.6 (cationic) 3.0 (anionic) 2.0 (neutral)	Free drug suspension (processed without lipids)	[35]
BCS class IV drugs							
Curcumin (herbal supplement)	SPC:SDC (85:15 *w*/*w*) Coating with Silica	93.3%	Liquid: liposome dispersion	Rat	2.3 (uncoated); 3.3 (silica-coated)	Free drug suspension	[36]
	SPC:CH:TPGS:drug (20:2:12:1) Coating with TMC	86.7%	Liquid: liposome dispersion	Rat	6.7 (uncoated); 10.6 (TMC-coated)	Free drug suspension	[37]
	SPC:SDC (70:25 *w*/*w*) Coating with TMC and CMCS	ND	Liquid: liposome dispersion	Rat	6.3 (CMCS/TMC-coated); 2 (TMC-coated)	Uncoated liposomes	[38]
Cyclosporine A (immunosuppressant)	ePC:Cremophor EL (10:0.5)	96.3%	Solid: proliposomes (lactose)	Rat	9.6	Free drug suspension	[39]
	SPC:SDC (3:1)	94.0%	Liquid: liposome dispersion	Rat	1.2 (NS)	Sandimmune Neoral^®®^	[40]
	SPC:CH (20:1) Coating with OACS	98.0%	Liquid: liposome dispersion	Rat	1.7 (uncoated); 3.4 (OACS-coated)	Free drug suspension	[41]
	EPC:CH (28:5) with Pluronic F127	90.0%	Liquid: liposome dispersion	Rat	1.8	Unmodified liposomes	[42]
Daidzein (natural compound)	SPC:CH:DSPEPEG2000 (55:40:5)	80.2%	Solid: freeze dried liposomes with 3% sucrose	Rat	2.5	Free drug suspension	[43]
Lopinavir (antiviral)	HSPC, CH (7:3)	Approx. 89%	Solid: proliposome (mannitol)	Rat	2.2	Free drug suspension	[44]
Paclitaxel (anticancer)	SPC:CH:SA (24.5:11.5:2 *w*/*w*) Coating with PAA and then PAH	81.3%	Solid: freeze dried liposomes with mannitol	Rat	4.0 (double-layer coated)	Free drug suspension	[45]

**Table 2:** Major liposome-specific characteristics.

Characteristics	Representative Techniques
Particle size and size distribution	Dynamic light scattering (DLS), Electron microscopy
Morphology, lamellarity	Electron microscopy
Surface charge	Zeta potential analysis
Encapsulation efficiency	Separation of free drug (dialysis, ultrafiltration, size exclusion chromatography) and drug analysis (HPLC etc.)
Release rate	Release in physiological media or storage buffer
Physical stability	Particle size change in physiological media or storage buffer

**Table 3:** Advantages and disadvantages of liposomes as an oral delivery system.

Advantages	Disadvantages
Biocompatibility	Physical instability in liquid state
Versatility for drug encapsulation	Lysolipid formation by chemical degradation
Flexibility of membrane components	Drug leakage
Capability of surface modification	Disruption in the stomach
Proposed enhanced permeation	Low permeability of intact liposome in the GI tract
Modifiable pharmacokinetic behavior	Difficulty in mass production and quality control

**Table 4:** Pharmacokinetic parameters of curcumin in rats from different studies.

Researchers	Formulations	Dose	F	AUC_0–∞_ (ng·h/L) (* AUC_0–12h_)	C_max_ (ng/L)
Li et al., 2012	Free drug suspension	50 mg/kg (oral)	-	86.65 *	71.35
	Liposomes (SPC:SDC)	50 mg/kg (oral)	-	203.64 *	128.78
	Silica-coated liposome	50 mg/kg (oral)	-	673.79 *	446.66
Chen et al., 2012	Free drug suspension	250 mg/kg (oral)	-	244,770	46,130
	Liposomes (SPC:CH:TPGS)	40 mg/kg (oral)	-	263,770	32,120
	TMC-coated liposomes	40 mg/kg (oral)	-	416,580	35,460
Tian et al., 2018	Liposomes (SPC:SDC)	10 mg/kg (oral)	6%	528,900 *	48,200
	TMC-coated liposomes	10 mg/kg (oral)	12%	1,218,200 *	78,300
	CMCS/TMC-coated liposomes	10 mg/kg (oral)	38%	3,021,200 *	167,800
Wang et al., 2020	Intravenous	40 mg/kg (i.v.)	-	268,900	-
	Commercial product 1 (tablet)	250 mg/kg (oral)	0.9%	20,000	12,600
	Commercial product 2 (capsule)	250 mg/kg (oral)	0.6%	10,740	9920
	Powder (Sigma)	250 mg/kg (oral)	3.1%	45,600	17,800

**Table 5:** Pharmacokinetic parameters of cyclosporine A in rats from different studies.

Researchers	Formulations	Mean Diameter	Dose	AUC_0–∞_ (µg·h/mL)	C_max_ (µg/L)
Shah et al., 2006	drug suspension	-	10 mg/kg	0.2253	0.09
	EPC/CreEL-proLip	10.34 µm	10 mg/kg	2.155	0.3
Guan et al., 2011	Microemulsion	-	15 mg/kg	65.41 ± 29.55	2.57 ± 0.20
	SPC/SDC Lip	85.6 nm	15 mg/kg	73.90 ± 6.63	2.65 ± 0.70
	SPC/CH Lip	98.1 nm	15 mg/kg	60.49 ± 10.79	2.67 ± 0.69
Chen et al., 2013	EPC/CH Lip	165.25 nm	10 mg/kg	9.18 ± 1.06 *	1.14 ± 0.23
	PF127-Lip	172.82 nm	10 mg/kg	11.59 ± 0.70 *	1.37 ± 0.15
	CS-Lip	207.81 nm	10 mg/kg	6.30 ± 0.97 *	0.79 ± 0.10
Deng et al., 2015	drug suspension	-	15 mg/kg	31.14 ± 1.30	1.10 ± 0.14
	Microemulsion	-	15 mg/kg	69.34 ± 7.93	3.40 ± 0.24
	SPC/CH Lip	58.94 nm	15 mg/kg	53.29 ± 4.59	2.85 ± 0.16
	OACS-Lip	69.12 nm	15 mg/kg	100.98 ± 13.08	4.14 ± 0.26

Lip: liposomes; CreEL: Cremophor EL; * AUC_0–12h._
